# Thinking Quantitatively of RNA-Based Information Transfer via Extracellular Vesicles: Lessons to Learn for the Design of RNA-Loaded EVs

**DOI:** 10.3390/pharmaceutics13111931

**Published:** 2021-11-15

**Authors:** Max Piffoux, Jeanne Volatron, Amanda K. A. Silva, Florence Gazeau

**Affiliations:** 1 Centre Léon Bérard, Department of Medical Oncology, 69003 Lyon, France; 2 INSERM UMR 1197-Interaction Cellules Souches-Niches: Physiologie, Tumeurs et Réparation Tissulaire, 94800 Villejuif, France; 3 Laboratoire MSC Matière et Systèmes Complexes, Université de Paris, CNRS UMR 7057, 75006 Paris, France; 4 Centre Hospitalier Lyon-Sud, Medical Oncology, Institut de Cancérologie des Hospices Civils de Lyon (IC-HCL), CITOHL, 69002 Lyon, France; 5 EVerZom, 75014 Paris, France; jeanne.volatron@everzom.com

**Keywords:** extracellular vesicles, exosome, RNA, miRNA, mechanism of action, engineering, loading, targeting

## Abstract

Extracellular vesicles (EVs) are 50–1000 nm vesicles secreted by virtually any cell type in the body. They are expected to transfer information from one cell or tissue to another in a short- or long-distance way. RNA-based transfer of information via EVs at long distances is an interesting well-worn hypothesis which is ~15 years old. We review from a quantitative point of view the different facets of this hypothesis, ranging from natural RNA loading in EVs, EV pharmacokinetic modeling, EV targeting, endosomal escape and RNA delivery efficiency. Despite the unique intracellular delivery properties endowed by EVs, we show that the transfer of RNA naturally present in EVs might be limited in a physiological context and discuss the lessons we can learn from this example to design efficient RNA-loaded engineered EVs for biotherapies. We also discuss other potential EV mediated information transfer mechanisms, among which are ligand–receptor mechanisms.

## 1. Introduction

Extracellular vesicles (EVs) are sub-cellular entities delineated by a lipid bilayer, containing biomolecules from parental cells, released either spontaneously or after induction. EVs are subcellular entities that partly reflect the composition of their parental cells, containing a portion of parental cell cytosol (proteins, RNA, and even organelles, etc.) encapsulated by a bilayer membrane with membrane proteins, lipids, etc. EVs contribute to intercellular communication by delivering a variety of bio-molecule cargo like nucleic acids, proteins, and lipids that modify the recipient cells. Their composition depends on the mother cells and on the environmental cues triggering EV secretion. Extracellular vesicles are usually described in three common subtypes: exosomes are 50–200 nm entities produced in multivesicular bodies and secreted after fusion with the plasma membrane, microvesicles are 100–1000 nm vesicles shed directly by the plasma membrane by budding and apoptotic bodies are 50–5000 nm objects secreted specifically during apoptotic cell death. All these objects are found mixed together in biofluids, and most of the time are called by the generic term extracellular vesicles, as our ability to specifically enrich or purify and characterize a specific subset is very limited, leading to complex mixtures in most cases. This terminology is strongly recommended by the International Society of Extracellular Vesicles [1]. Although some markers have been proposed, well-accepted specific markers are still lacking to distinguish these vesicle sub-populations [1]. A key issue in this regard is the intrinsic nature of EVs that display a lot of non-specifically enriched as well as targeted proteins. In fact, most if not all parental cell constituents were found in their derived EVs when EV signatures were compared with their parental cell with -omics methods (proteomics, lipidomics, RNAseq) [2]. EVs raised a particular interest in the community when they were reported to have therapeutic activities in a myriad of preclinical models ranging from cardiovascular, neurodegenerative, skin, COVID-19 pneumopathy, inflammatory diseases and regenerative medicine via the induction of various pleiotropic effects: angiogenesis [3], immunomodulation [4], cell proliferation [5], inhibiting fibrosis [6], resolving inflammation [7], etc. Hence, a growing number of clinical trials are launched based on the therapeutic properties of EVs [8]. However, the modes of action of EVs could not be reduced to one single component that could drive their therapeutic activities. 

The hypothesis that EV’s main activity could be due to RNA transfer has been raised in the seminal publication from Valadi et al. [9] in 2007. In this article, the authors convincingly show that EVs purified from murine cell lines contain small size RNAs, and that these RNAs may be found in human cells after exposition to EVs. The translation of these RNAs in protein and its relevance in physiology is more subject to caution as it is only based on a proteomic screening that reported the detection of three murine proteins (not detected in all samples) in lysate from human cells (incubated with murine EVs) that were originally not present in the EV preparation (only 271 protein detected in EVs). Of these three, one was not detected in EV’s RNA. Due to intrinsic limits in the sensibility and specificity of non-targeted proteomics, it is possible that this small signal may be a false positive, and more importantly that this transfer is due to the use of high and non-physiologic concentrations of EVs that do not mirror the in vivo environment (8:1 producing/recipient cell ratio). 

Since then, a vast sum of articles has been published to describe how EVs may be a natural vector for information transfer through RNA [10] and how their therapeutic activity in diseases may be mediated by RNAs. 

As in the study by Valadi et al. [9], the presence of a particular RNA is usually described in the EV preparation, and a therapeutic effect is described in vitro or in vivo. The link between the two is usually limited to a correlation and/or demonstrated in vitro, but the experiments performed do not allow to conclude on causality in vivo. In this review, we will discuss the available evidence of in vivo long-distance transfer of RNA via EVs and its physiological relevance. 

The field also benefited from the interest of researchers at the intersection between three emerging and attractive domains: RNA and si/miRNA biology, drug delivery and extracellular vesicles’ biology. EVs were subsequently discussed as potential interesting biogenic nanovectors for RNA as an alternative to synthetic vectors with specific advantages. Compared to synthetic lipid nanoparticles, EVs’ intrinsic “biological nature” might be responsible for their limited toxicological profile, low immunogenicity, extravasation in tissue, crossing biological barriers like the blood–brain barrier, the ability to target specific cell types, the ability to fuse with cell membrane with exceptional endosomal escape and cargo delivery. A substantial amount of papers later on described the use of EVs to deliver various drugs ranging from small molecules like chemotherapies to complex biomolecules including proteins, receptors or nucleic acids (miRNA, siRNA, plasmids, etc.) [11,12]. However, the mechanisms regulating RNA loading in EV as well as their transfer and delivery to acceptor cells are not yet elucidated [13]. Most of the works that aimed to quantify EV-mediated RNA information transfer were done in simplified set-up, mostly in vitro. A limited amount of papers interrogate the efficiency of EV-mediated RNA transfer in vivo in relevant physiological models. In 2012, Dr Eugene D. Sverdlov published a critical opinion paper [14] questioning: (1) whether data on RNA transfer obtained in vitro in the literature are physiologically relevant in terms of molar concentration, and (2) whether the potential of exosomes and other EVs to deliver information (e.g., through nucleic acids, specific targeting or enhanced endosomal escape) is physiologically relevant. These issues are also of critical importance when considering the design of EVs or the use of cell secretome vesicular fraction to deliver targeted RNA in a therapeutic purpose. Serdlov’s paper proposed an original physical chemistry-oriented quantitative point of view on the EV field. He argued citing Karl Popper’s falsification principle that a theory cannot be demonstrated if there is no way to falsify it. Considering the hypothesis of physiological RNA transfer through EVs, it means that it could hardly be demonstrated without specific activators and/or inhibitors of EV secretion (i.e., only affecting EV secretion and no other side-effects). Today, the quest for selective inhibitors and activators of EV secretion is still ongoing, with interesting molecules claimed to be selective [15], whereas they target very common biological processes such as farnesylation, or present the well-described side effects of antifongics, antibiotics or other drugs [16,17,18]. In this review, we aim at providing a bio-physico-chemical quantitative point of view on long-distance EV-mediated information transfer via RNA transfer in physiological settings based on new data produced since 2012 in the field. We also discuss in this context the data obtained in therapeutic settings with natural EVs and engineered EVs and compare it to RNA synthetic vectors like lipid nanoparticles.

## 2. Claiming a RNA-Based Mechanism of Action for Native EVs?

Many teams reported an (mi)RNA-based mechanism of action for EVs. Most proofs of concept are however in vitro, and in vivo data are relatively scarce or not designed to claim an RNA based mechanism of action delivered via EVs at long distances and/or limited to short-distance transfer (Table 1):

- In glioblastoma (GBM), Abels et al. describe short distance communication through EVs from tumor cells to microglia to induce microglia reprogramming. The presence of EVs was detected in 0.3% of microglial cells, and the presence of the miRNA of interest transferred by EVs was detected in these sorted 0.3% of cells. However, no clear target protein silencing was found (only 4 out of 59 validated targets). It may be possible that the miRNA detected would partly be coming from the retention of EVs (and its associated miRNA) in endosome of microglial cells, without intracytosolic delivery. Furthermore, to clarify that the mechanism was mediated by EVs, they injected EVs in mice brains, but the dose used (1.26 × 10^9^ EVs in the mouse striatum, i.e., about 6 × 10^6^ cells or 210 EVs/cell) is highly supra-physiologic compared to what was detected in the first experiments (0.3% of cells containing EVs) [19].
- Lucero et al. demonstrate the short distance effect of glioblastoma-derived EVs to induce angiogenesis via miRNAs in vitro, and claim it to be also valid in humans only based on a correlation with a human glioblastoma transcriptomic “fingerprint” [20]. However, no clear demonstration of causality is proposed.
- Shen et al. demonstrate the effect of EVs derived from tumors to induce stemness via miRNA in surrounding cells in vitro (at supra physiologic doses) and claim it to be also valid in vivo in tumor-bearing mice. However, they used Rab7 KO tumors as a control to inhibit EV production, a KO that also has a lot of other side effects [21]. It is therefore difficult to know whether this effect is mediated by EVs and by the miRNA inside them.
- Ying et al. demonstrate a role for miR-155 transferred by EVs in vitro in glucose tolerance and use an elegant system of bone marrow transplantation to investigate the role of hematopoietic derived miR-155 in a KO mouse. They later claim that the partial rescue of physiologic glucose tolerance is mediated by EVs in vivo although it may also be mediated by other intercellular transfer mechanisms like tunneling nanotubes (TNT), especially to transfer at short distance miRNA from a very macrophage-rich organ like liver to surrounding hepatocytes [22]. The same miRNA-155 has indeed been shown to be able to be transferred through TNT [23].
- Chen et al. claimed that miR-375 overexpressing EVs were able to promote bone regeneration but the effect in vivo is not significantly different from the EV control group [5].
- Thomou et al. help us to raise other non-trivial questions on vesicular versus non vesicular mediated RNA transfer. He proposed that EVs from adipose tissue would be able to transfer miRNA to liver cells and induce RNA silencing in vivo. The protein expression is reduced by up to ∼95% after injection of serum-derived EVs (from donor mice with brown adipose tissue expressing the miRNA of interest) to miR-KO mice [24]. Strictly speaking, the demonstration proves that a serum factor purified with common EV purification protocols from the donor mice leads to specific miRNA-mediated silencing in mice. It raises the question of whether this effect may be at least partly mediated by an extra-vesicular miRNA in serum co-purified with EVs. Chevillet et al. showed that only ∼2.5% of miRNA extracted by common EV purification methods were indeed loaded in EVs [25], leaving 97.5% of them outside, mostly stably complexed with the Ago2 to form the “RNA-induced silencing complex” (RISC) [26]. However, intriguingly, no data to our knowledge reported the potential effect of non-vesicular miRNA mediated effect on receptor cells and even less side to side comparisons with vesicular miRNA, leaving this question unsolved for the moment.
- Other teams claimed the demonstration of an efficient transfer of CRE-mRNA via EVs [27,28,29] in vivo. This highly sensitive “on/off” system induces or stops the expression of a particular fluorescent protein upon delivery of the CRE-recombinase protein or its RNA. Although it is very different from a physiologic system, it may still be of interest as a proof of concept. However, this assay has shown limited transduction efficacy even with a high dose of EVs (e.g., in Ilahibaks et al. [30] achieved ∼15% transduction efficacy by ∼8300 EV/cell in vitro, i.e., *intra*-*cytosolic* transfer of at least one CRE protein or RNA). More importantly, it may be biased by the transfer of a single CRE recombinase protein (instead of CRE-mRNA) from the donor EVs, although it was not detected in these articles. On the contrary other teams clearly reported the presence of CRE protein in EVs produced from CRE-producing cells [31]. Nevertheless, the correlation between an observed new phenotype and the CRE recombination does not mean causality between these two facts. As an example, a particular cell with a particularly elevated phagocytosis would be particularly sensitive to EV endocytosis and CRE recombination (as seen by the fluorescent protein expression) compared to a “steady” cell from the same cell type. Differences between them would then be explained by their more or less elevated *pre-existing* phagocytosis phenotype even before EV absorption. Other authors proposed other potential biases in this kind of experiment, like the change of phenotype due to liposome/EV absorption by cells [32,33].

Although the in vivo relevance of RNA-mediated information transfer via EVs lacks clear demonstration in physio-pathological settings, plenty of proofs-of-concept are described in vitro with efficient silencing efficacies [20]. This may be partly explained by the fact that stoichiometric analysis to use physiologically relevant EV concentrations is usually not considered, leading to a typical in vitro EV/cell ratio of about >150,000 EVs/cell [20]. Secondly, for both in vitro and in vivo data, most reports are using cell lines KO for a specific miRNA as a control, but this control might be questionable. Indeed, miRNA usually have about 90–300 targets [34,35], i.e., the comparative effect may be mediated by a lot of other non RNA-mediated effects due to cell physiology dysregulation. Another important factor to consider is the kinetic effect of EVs, which has rarely been investigated. In the few studies that we found, EVs mediated their effect within minutes (less than 60 min, peak effect at less than 20 min [36,37]), a kinetic that is not the one expected by RNA-mediated information transfer. 

Of note, apart from the classical “EV travel to the other organ” mechanism of information transfer to distant locations, this transfer may also happen via EV transfer at short distance to a circulating cell (e.g., T cells draining lymph nodes), that can later on reach other organs to exert an effect.

## 3. Physiological Effect of RNA Cargo in EVs: A Natural RNA Vector?

### 3.1. Stochiometric Evaluation of RNA Loading in EVs

Sverdlov claimed that it is very unlikely that naturally circulating EVs transfer a significant part of information through RNA in vivo at long distances in physiological states [14]. He argued that the best candidates for information transfer would be self-amplifying (e.g., mRNA) and/or have a regulatory function (e.g., a transcription factor, a miRNA). At the time, he made the hypothesis that RNA inside EVs was not subject to strong selection. Baglio et al. [38] and other groups found that most RNA in various types of EVs (from tumor, MSCs, immune cells and serum, isolated by various methods (ultra)-centrifugation or affinity column) were small <400 nucleotides (nt) long RNA [39,40,41,42]. Among them, most are tRNAs (that can hardly be expected to have an effect) and miRNA only constituted ∼0.9% [43] of RNA reads. Although the miRNA are relatively enriched (∼10 fold compared to cell RNA^4^), enrichment may largely be due to the nonspecific size selection biased to the smaller sizes such as tRNAs. As an example, 16 S RNA (1,6 kB), a typical medium-size RNA has a hydrodynamic diameter of ∼30 nm [44], whereas miRNA (20–83 nts) have a cylinder shape with a 2 nm diameter and a 7–20 nm length. mRNA encapsulation inside EVs also depends on their local concentration around EV formation sites, as well as mRNA interaction with membrane lipids and proteins [45]. Before being functional, miRNA are getting through the pri- and pre-miRNA state. To be potentially active if they get to the target cell cytosol, miRNA needs either (i) to be not yet associated with Ago2 to form the RISC complex but still able to bind to it (i.e., being pri- or pre-miRNA) and therefore they would be able to bind it later on in the recipient cell cytosol or (ii) to already be associated with the RISC complex as a miRNA, a state in which they can exert their silencing activity directly. Importantly, association of miRNA to the RISC complex allows them to be much more stable than if left alone where it can be rapidly degraded by nuclease, in particular in the context of EV travel through endosomes (containing nucleases) in the target cell. Interestingly, as reported by Chen et al. [39], most of miRNA in EVs derived from mesenchymal stromal cells (MSC) were pri- and pre-miRNA, but not mature ones (coupled to RISC). Furthermore, as discussed in an opinion paper from the same team in 2018 [43], the presence of RISC is hardly found inside EVs produced in vitro in most types of EVs (28 occurrences of DICER and 31 of Ago2/EIF2 C2 over 349,988 protein entries in Vesiclepedia [46] on the 10 August 2021) and not present in MSC-EVs [43,46], a finding which was confirmed in our team (Ago2 was found at very low level in MSC-EVs). Lastly, co-purification of extra-vesicular miRNA fractions, mostly reported to be associated with RISC [47], may partly explain the results observed. Altogether, this means that miRNA in EVs may potentially exert a silencing activity once delivered to the cytosol through binding target cell RISC complexes, but may face an obstacle course to do so by surviving endosomal and cytosolic nucleases and competing with the target cell miRNA pool to be part of the RISC complex. 

Sverdlov proposed a rough approximation of the maximal amount of RNA per EV if they are densely packed in EV of 100 nm diameter: ∼1600 RNA/EV for 1000-nt RNA and ∼6700 RNAs/EVs for 200 nts RNA. However, when measured by total RNA quantification [48], the number of RNA per EV was less than one in serum-derived EVs. Another team reported the presence of ∼7 µg of RNA per 10^10^ EVs dosed by bulk representing ∼6500 RNA molecules per EV [43], but the presence, as discussed by the authors, of contaminating surrounding extra-vesicular RNA may artificially enhance this number. As an example, once extra-vesicular RNA is removed from serum-derived EV preparations (using differential centrifugation and size-exclusion chromatography) only ∼2.5% of total miRNA remains in the serum-derived EV fraction [25,26,49,50,51,52]. Most of the time, purification strategies used are not allowing complete extra-vesicular RNA removal (in particular in serum where it represents a large fraction of RNA), therefore attribution of a particular effect to intra-vesicular EVs may be difficult. Quantitative results on the amount of miRNA per EVs estimates that most represented miRNA can hardly be found in 1 out of 100 exosomes (the range varies for each miRNA from one copy per 9 exosomes to one copy per 47,162 exosomes, mean of 1 copy per 121 exosomes using digital PCR, a reliable and sensitive quantitative method) [25]. Knowing that they detected 131 miRNA in total, the estimated miRNA per EV should be considered to be ∼1 per EV. Chen et al. [43] use a different approximation to conclude that MSC EVs contain ∼1.3 miRNA per EV, and one particular miRNA in only ∼1% of EVs. Knowing that miRNA represent ∼0.9% of RNA reads in EV RNA deep sequencing [53], one can expect that about 100 RNAs are present in each EV, i.e., much less than if densely packed (∼6700 small RNAs). 

Although miRNA levels in EVs seem too low and not packaged in order to be effective, one can expect that other coding mRNA may explain the therapeutic effects mediated by EVs, especially if they are protected by EVs from serum RNAse. Once again, quantitative analysis gives us insights into this hypothesis. Even more than for miRNA, RNA amount in EVs is very low and biased toward small RNAs (<300 nts) that are mostly fragments of degraded mRNA (a typical mRNA measures ∼2000 nts [54]). 

Altogether, it indicates that mRNA can hardly be a good candidate for EV mechanism of action. This data review gives a glimpse of the natural amount of RNA molecules (∼1 miRNA, ∼100 small RNAs) in EV cargo, an unexpectedly limited one. Hence, in the therapeutic use of EVs relying on RNA transfer, one should not count on naturally present RNA molecules in EVs. Instead, efficient engineering method are needed to optimize the loading of RNA in EVs. 

### 3.2. Navigating the Bloodstream and Getting to the Target?

Once loaded with RNA, in order to exert an effect at a significant distance, EVs have to get inside the bloodstream to reach other tissues. If not produced directly inside the bloodstream, they are secreted in interstitial fluid to later on get to the bloodstream. The main physiological barrier to the long-distance travel of EVs from organs is probably their limited ability to get to the bloodstream. There is a probably significant recapture by surrounding cells in the interstitial fluid (ISF) before getting to the bloodstream. Indeed, the amount of interstitial fluid is expected to represent about 16% of the body weight (11 L in a 70 kg adult), and the concentration of EVs in ISF is on overall ∼12 times more concentrated than in plasma [55]. The ISF flow from interstitial fluid to plasma was calculated to be about 2.9 L/24 h [56] meaning that EVs may spend a significant time in ISF. Although the half-life of EVs in blood is relatively well estimated (∼3–15 min), it is not known in ISF, but it is probably in the same order of magnitude (10 min). Therefore, if about 1/4 of ISF goes to the bloodstream every day, the turnover of ISF (∼96 h) is far longer than the typical EV half-life in ISF, meaning that most EVs (96 h × 60/10 = 576 EV half-lives) are recaptured inside the tissue before reaching the bloodstream. 

A simple model taking into consideration key EV pharmacokinetic parameters helps to get an idea of what happens in physiological conditions for EVs navigating in the bloodstream. Indeed, the amount of EVs received per cell from blood per 24 h can be estimated by the following Equation (1):

(1) $$\frac{\frac{EV}{Cell}}{day}=\frac{Vol\left(plasma\right)\times Ctot\left(EV\right)\times f\left(EV\;subtype\right)\times \frac{Ln\left(2\right)}{\tau ½}\times t\times f\left(target\;tissue\right)}{Nb\;Cell\left(tissue\right)}$$

where *Ctot*(*EV*) is the total concentration of EVs in the blood (in EV/L), *Vol*(*plasma*) is the volume of plasma in the organism (3 L for humans), *f*(*EV subtype*) is the fraction of a particular EV subtype of interest, τ½ is the EV half-life in the blood stream (in minute), *t* is the number of minutes per day (1440), *f*(*target tissue*) is the fraction of EVs that target a particular tissue of interest and *Nb Cell*(*tissue*) is the number of cell in the tissue of interest. Of note, this model does not take into account the excretion of EVs in urine and other biofluids as it is considered to be negligible in biodistribution studies [57]. Furthermore, this model is only valid for steady states with EV generation and recapture balance being relatively stable and therefore does not apply to bolus injections of EVs. An estimation of relevant parameters to consider is provided in Table 2, some of them are estimated from mouse data (e.g., half-life), the model suffers a lot of approximations but yet gives an interesting approximation in terms of order of magnitude.

It has to be noted that the limited half-life calculated in mice is usually longer in humans [63] for similar compounds, but this half-life increase would diminish the amount of EVs received per cell per day in our equation. The attraction toward reticulo-endothelial-system rich organs (liver, spleen) means that a large fraction of EVs are captured by a non-specific capture mechanism (EVs are mostly present in Kupffer cells in the liver [64]). Of note, EVs produced in the body in presence of opsonins seem to behave similarly in biodistribution compared to ex vivo produced EVs [57,65].

Using this equation, the mean total amount of EVs received from blood from all kinds of parental cells by all cells of the organism is estimated to be ∼4.3 × 10^14^/day, the mean number of EVs received per cell is ∼11.5 although it largely varies from an organ to another, e.g., is ∼1069/day per liver cell and 0.7/day per brain cell. On another side, significant variations occur depending on the subtype of interest considered: contrary to a quite common vision, most (99.8%) of EVs in the blood come from hematopoietic cells whereas only 0.2% of them come from non-hematopoietic tissues, most of them (81%) coming from adipose tissue. Therefore, the distant effect of a particular organ (or a tumor, see Box 1) toward another via EVs may be considered limited by the number of EVs exchanged, e.g., all non-hematopoietic tissues collectively only send ∼2 EVs per day to each liver cell and collectively ∼0.001 EV per day to each brain cell. It has to be noted that these 4.3 × 10^14^ EVs secreted in the blood per day represent collectively a mass of ∼0.39 g (for 120 nm vesicles, larger objects represent a very limited fraction of objects), a relatively small mass compared to the number of cells that die every day in our body, i.e., ∼10^11^ per day [66], representing ∼190 g per day (for a 70 kg adult with 3.72 × 10^13^ cells, although a fraction may rather be lost by desquamation and not transferred to surrounding cells) that would then be transferred by apoptotic bodies to surrounding cells *at a short distance*. Most cells in the body in physiologic conditions die and transfer their content to the surrounding cells via apoptosis and subsequent apoptotic bodies, one of the subtypes of EVs. Interestingly, this comparison gives an insight on the relative importance of both long distance communication via EV (∼0.39 g of tissue transferred from one cell to another) versus short distance communication inside tissues (or lymph) that do not usually reach the bloodstream (representing ∼100 g of tissue transferred from one cell to another). It also shows the relative importance of relatively small EVs found in the bloodstream (mostly exosomes and microvesicles) versus the amount of small and large EVs (especially apoptotic bodies) exchanged inside tissues. More details on the quantitative data on RNA transfer at short distances are discussed in Box 1.

Special attention should be paid to the short estimated EV half-life compared to most hormones, proteins and drug delivery systems. Indeed, apart from being an interesting parameter for modelization, it is also an important driver to control the potential specific targeting of a tissue/cell type by EVs. Indeed, the whole blood volume circulates ∼3 times/min [67], and therefore may get through the cerebral circulation and potentially interact with brain cells only ∼7 (half-life) × 3 × 0.15 = 3.15 times (cerebral blood flow is ∼700 mL/min [68], representing ∼15% of blood circulation). As discussed before, the very limited half-life and massive attraction toward reticulo-endothelial-system rich organs (liver, spleen) mean that a large fraction of EVs is captured by a non-specific capture mechanism. This is consistent with the limited accumulation of brain-targeted EVs (with RVG peptide) compared to non-targeted ones that increased brain targeting from 0.5 to 1% of the total EV dose injected IV [57]. In contrast, PEGylated liposomes with a 48 h half-life would get through the brain circulation ∼2880 × 3 × 0.15 = 1296 times (leaving much more chance to interact specifically with a targeted receptor of interest), although blood–brain barrier (BBB) crossing efficacy later on by EVs and liposomes may be totally different. Indeed, Banks et al. reported that EVs detected in mice brains after systemic injection (∼1% of total dose [57]) were located at 58–92% in the parenchyma depending on cell type [69], indicating a very good ability to cross the BBB. They also reported a significant efflux from the brain to the blood (half-life of ∼7 min).

Box 1 **Quantitative data on short distance RNA transfer.**

Haimovich et al. [70] recently described the first quantitative estimation of RNA transfer between cells at short distances, although it was made in vitro. They used fluorescent RNAs to investigate their transfer from one cell to another and discovered that this transfer goes up to 4% of the amount of a specific RNA pool, but is usually lower. Interestingly, this was described in 2 D classical cultures; this may be very different in 3-D cultures but no quantitative data are available up to date. Of note, the authors claim that the transfer is mediated in a vast majority by tunneling nanotubes and exclude a substantial effect of EVs by describing a very limited transfer if donor and recipient cells are separated using a transwell plate, physical separation or purification of EVs followed by incubation with recipient cells. We believe the demonstration of the limited role of EVs not to be appropriate as the local concentration at the surface of recipient cells is much lower than the in vivo concentration of EVs in tissues. Indeed, the usual EV concentration in cell cultures after 24 h incubation is very low: in the ∼10^8^ EV/mL range (personal data), whereas the concentration in plasma is ∼10^9^ EV/mL and concentration in ISF is ∼12 fold higher, i.e., probably ∼1.2 × 10^9^ EV/mL [55]. Of note, the potential effect of EVs’ containment in ISF between cells and the effect of physical forces acting on tissues may even favor interaction with cells and favor even more RNA transfer via EVs in vivo compared to in vitro.

Altogether, these assumptions give a quantitative insight on how difficult the long-distance trip of a particular EV may be to a particular cell of interest in a distant organ. Once in the bloodstream, long-distance travel of EVs is mostly limited by their non specific capture and subsequent elimination by RES, and this has proved true for all kinds of EVs tested from hematopoietic origin or not [57,65]. However, this estimation may be different in pathological conditions in which injured organs, inflammation sites or tumors might be a significantly enhanced source of EVs but also a sink for circulating EVs (see Box 2). 

### 3.3. A Very Interesting Intra-Cytosolic RNA Delivery (Endosomal Escape)

Once EVs reach an acceptor cell, the way they are internalized and the mechanisms by which they deliver their cargo are still not fully clarified. De Jong et al. reported with an interesting CRISPR-based system that delivery was partly dependent on micropinocytosis (in particular Pak1, Rak1) or endocytosis (in particular Cav1 and RhoA) [13]. It is now well accepted that intra-cytosolic delivery depends on acidic endosomal escape [71]. Although data are very scarce, the reported EVs endosomal escape efficacies vary from 10% after 2 h to 24.5% after 12 h in Joshi et al. [72] and about 20–30% in Bonsergent et al. [71] Although direct fusion with the plasma membrane is possible, it is expected to represent a much smaller fraction of intra-cytosolic delivery when looking at the delivery kinetic. These numbers are to be compared with the natural endosomal escape that is reported to be about 2–7% depending on the cell type [73], about 0.1–2% for synthetic vectors [74] and about 40–50% for viral vectors like AAVs [75]. 

### 3.4. Is the Physiologic RNA in EVs Dose Sufficient to Achieve an Effect?

Preceding discussions show us that once a vesicle of interest, on average containing ∼1 miRNA (among about 100 different ones) and ∼100 small RNAs (∼200 nts) reaches a cell, it has about 20% chance to liberate its cargo. Taking into consideration the average number of natural EVs received per day in physiological conditions that vary from ∼0.7 (brain) to 1000 (liver) per cell (with a mean at ∼10), the amount of a specific miRNA received ranges from ∼0.014 to 2 per day (total amount of miRNAs per day ranges from 1.4 to 200). This number has to be considered relative to the reported number of miRNA/cell that varies from 115,330 copies in mice liver cells (200 miRNA out of 115,330, i.e., 0.17%) and 11,587 in hematopoietic stem cells [76]. Different teams reported that ∼2000 to 10,000 cytosolic siRNAs per cell were required to reach a correct inhibition efficiency [77,78]. Using the same simplified pharmacokinetic model, the total amount of RNA (∼200 nts) molecules reaching the cytosol from EVs varies from ∼140 to 20,000/day (versus 1.4–200 miRNAs). An estimated 360,000 mRNA are contained in a typical cell cytosol [79], and mRNA represents only about 1–5% of RNA in a cell (meaning that we would expect about 7.2–36 × 10^6^ RNA molecule per cell). In that case, one may see that the amount of RNA delivered in these conditions by EVs is small (from 0.0004 to 0.2% of total cell RNA). For comparison, it is estimated that the number of proteins *per EV* is ∼500–2000 proteins [80]. 

In line with the preceding discussion, the RNA-based mechanism of action (MOA) for effects mediated by non-modified native EVs in therapeutic conditions has previously been challenged by comparing it to data obtained from siRNA experiments. In most preclinical studies, EV doses usually range from ∼1 to 200 µg per mouse [81], corresponding to about 10^10^ to 10^12^ EV/mouse depending on EV preparation and dosage methods. If we consider ∼1 miRNA per EV, this dose represents ∼10^10^ to 10^12^ miRNA per dose, corresponding to about ∼0.2–20 ng of miRNA/mouse or ∼0.016–1.6 pmol/mouse. siRNA doses reported to be efficient in vivo in systemic injections are rather in the microgram range (27 to 750 µg/mouse [82,83]). One explanation is that the observed therapeutic effect of native EVs is not mediated by their naturally loaded (mi)RNAs. Indeed, this ∼10^3^–10^4^ fold difference was though too big to be explained by a very high difference in delivery efficacy [43,80]. However, this may be now discussed in view of recent results comparing engineered EVs to synthetic RNA nanovectors. 

Indeed, recently reported delivery efficacy of EVs obtained in vivo show a ∼10–300 fold improvement in favor of EVs [84] compared to lipid nanoparticles (although the authors discuss the estimation of miRNA concentration with their method may favor EV reported efficacy by ∼10 fold [85,86]). The authors used the natural ability of pre-miR-451 to be enriched preferentially in EVs and used it as a backbone to couple with an siRNA of interest in order to target it inside EVs [84]. They then used these engineered EVs to target the liver, intestine or kidney glomeruli and achieve various target knockdown. Interestingly, this ∼10 to-300 fold improvement in terms of RNA cytosolic delivery in favor of EV in vivo is fully consistent with independent data on delivery efficacies reported for synthetic vectors: EVs reach a ∼20% endosomal escape rate [71] compared to 0.1 to 2% for synthetic vectors [87], which leads to a ∼50 fold increased cytosolic delivery. Even higher differences (up to 10^4^) were reported in the delivery efficacy in favor of EVs in vitro [32]. Importantly, such a fold change also takes into account the very different endocytosis rate that favors EVs compared and synthetic vectors in vitro but not in vivo [88].

Box 2 **EV-mediated RNA transfer in pathological conditions.**

Quantitative data on EVs present in pathological conditions (increased secretion, particular targeting, half-life, etc.) are relatively limited. Here, we propose working hypotheses based on little literature data. Quantitative hypotheses are used here as a way to estimate a maximal limit of RNA transfer in pathological conditions and to discuss whether this upper limit may be sufficient to achieve a sufficient amount of RNA transfer at a long distance to achieve an effect. Loading of RNA in pathological conditions (inflammation, sepsis, tumors) may be different from physiological ones, but it should still recapitulate parental cell RNA with a bias toward small RNAs with a total amount of miRNA per EV of about 1 as the total concentration of RNA does not substantially change in most pathological conditions. Nonetheless, it may largely increase the specific amount of an inflammation-induced (mi)RNA of interest up to 15 fold [89]. We hypothesize that it could then represent up to ∼0.2 miRNA of interest per EV (i.e., 2 times more than reported for classical most concentrated miRNAs). We also consider that secretion in pathological conditions may be particularly augmented, like in inflammatory diseases where a particular type of EV subtype may be increased up to 2 fold for platelets, maybe more for other cell types, although the total amount of EVs is not particularly increased [90,91,92]. In cancer, no clear increase in the total amount of EVs was reported [93], and we estimate a maximal concentration of tumor-derived EVs might represent 1% of total EVs in plasma (equivalent to 5 times the sum of all non-hematopoietic cell-derived EVs). EVs half-life in the inflammatory context has not been reported to our knowledge, we estimate that a 2-fold decrease may be the maximal variation as such decrease in half-life is common for proteins in inflammatory pathologies (and this would increase the total amount of EV transferred using our simplified model). Biodistribution may be shifted toward inflamed zones in analogy with nanoparticle EPR (enhanced permeability and retention rate) effect [94] although no data are available to our knowledge for EVs, and EVs can furthermore benefit from specific targeting compared to liposomes in tumors or inflamed tissues [95]. We considered a 5 fold increase in inflamed tissues as a good approximation using this analogy. Tumors were shown to accumulate up to 18% of MSC-EVs [96] (rather 0.5–4% in our experience [97] and in other reported data [98]) and tumor-derived EVs seems to display a very similar distribution profile (±30%) compared to serum EVs [99]. We make the assumption that EV uptake and endosomal escape should be similar in a physiological and pathological context, although EV uptake might be biased towards a specific cell population.

In total, when taking into account most favorable estimations of each parameter, we can calculate the following estimations: (i) In the inflammatory context, ∼2000 platelet-derived EVs/cell/day would reach liver cells (∼4 fold increase), and lead to the delivery of a maximal ∼80 miRNA molecules of interest in the cell cytosol (40 fold increase compared to non-inflammatory state) compared to an estimated >2000 copies of a specific miRNA needed to exert an effect [77,78], (ii) in the inflammatory context, ∼3.6 platelet-derived EVs/cell/day would reach brain cells (∼10 fold increase), and lead to the delivery of a maximal ∼0.14 miRNA copies of interest in the cell cytosol (100 fold increase compared to non-inflammatory state), (iii) in the oncological context, ∼10 tumor-derived EVs/cell/day would reach liver cells, and lead to the delivery of a maximal ∼0.4 copies of a miRNA of interest. Regarding total RNA, as discussed before, the picture stays the same with the delivery of <0.01% of the total amount of RNA in a cell. Based on these very controversial estimations, even in most favorable situations, we consider that RNA-based information transfer at a long distance may also be a relatively non-efficient mechanism in pathological settings when we consider naturally secreted and -circulating EVs. In contrast, the impact of the pathological environment may play an important role on the “adoptive” administration of natural or engineered EVs (increased homing to inflammation sites, accumulation in the liver with potential immunologic effect, increased penetration in tissues or uptake by specific cells).

**Figure 1:**
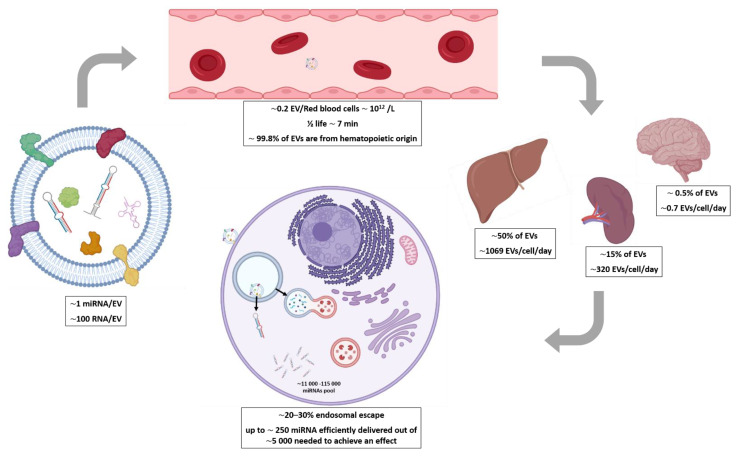
Summary of key numbers about RNA transfer via EVs in physiological conditions.

In conclusion, the aforementioned difference (∼10^3^–10^4^) in terms of the dose delivered to obtain an effect in vivo between siRNA delivered by synthetic vectors and miRNA in EVs may therefore be at least partly explained by the reported very good delivery efficacy of EVs (∼10–300 fold improvement), although other mechanisms of action might also contribute. Altogether, these quantitative estimates show (Figure 1) that distant communication by EVs via RNAs probably has limited efficacy in physiological conditions, although it may be a bit different in pathological conditions (see Box 2) and in the therapeutic use of EVs that are engineered to load large amounts of specific RNA.

## 4. Considerations on RNA Based Information Transfer in Therapeutic Settings 

### 4.1. Considerations on the Therapeutic Effect of RNA from Unmodified EVs 

RNA delivery has been reported to be the mechanism of action of natural EVs administrated for therapeutic purposes like MSC-EVs [100]. The main difference compared to naturally circulating EV is that the injected concentration in the specific EV of interest will be highly increased compared to their physiological concentration. For example, MSC-EVs are expected to represent much less than 0.1% of serum EVs [60], i.e., <10^9^ out of 10^12^. Using the previously described model, if we consider a human therapeutic dose of 10^12^ to 10^13^ EVs, one may expect that ∼2.5–25 EVs and ∼0.5–0.5 intra-cytosolic copies of an miRNA of interest would be delivered to each liver cells, or ∼0.75–7.5 EVs and ∼0.015–0.15 intra-cytosolic copies of an miRNA of interest to each cell in the spleen (an organ that contains a lot of immune cells of interest for these therapies). Whether it is sufficient to explain the therapeutic effect of MSC-EVs seems once again unlikely in systemic administrations, apart from a very efficient RNA “cocktail effect”. Local administrations (we approximate that it would touch only 100 g of tissue, 1/650 of the total amount of cells of a human body, i.e., 5.7 × 10^10^ cells) of similar doses (10^12^–10^13^) would lead to the delivery of ∼18–180 EVs and ∼0.35–3.5 intra-cytosolic copies of an miRNA of interest to each cell. It seems also difficult therefore that unmodified EVs’ therapeutic effect may be mostly mediated by RNAs.

### 4.2. Considerations on the Effect of RNA from Engineered EVs

Although unmodified EVs may difficultly have an effect via their RNA cargo, EVs may be very interesting vectors for synthetic delivery of exogenously loaded RNA (mi/si/mRNA). Exogenously EV-loaded synthetic RNA delivery for therapeutic purposes have been reported to treat various pathological conditions [101,102]. The problem of efficient EV loading is still highly debated, in particular in the case of electroporation [103] and was recently reviewed elsewhere by our team and others [11,104]. Briefly, loading of nucleic acid in EVs in EVs have been reported with various techniques ranging from electroporation [103], EV destruction-reformation techniques (slicing, extrusion or sonication [105]), permeabilization via saponin, the use of commercial transfection reagents like lipofectamine [106], heat shock [107], pH gradient [108], etc. Importantly, most of these methods suffer from a low loading efficiency, potential substantial denaturation of EVs and whether these methods lead to intra-vesicular loading or extra-vesicular aggregation on EVs is usually not investigated clearly [11]. 

The major difference of engineered EVs compared to the above described example of MSC-EVs is the high enrichment in the (mi/si)RNA of interest, in theory up to ∼60,000 for densely packed siRNA (21 nt) in 100 nm EV (a highly unfavorable physical state), although some papers report unexpected loading of up to 1.8 × 10^5^ copies per EV [109] (probably due to electroporation induced siRNA aggregation). Of note, the distinction between intra-vesicular and extra-vesicular (aggregated) loading may be considered as non-relevant as some authors reported the efficacy of siRNA delivery with EVs electroporated in conditions that are prone to induce siRNA aggregation [102]. Loading via engineering of the parental cell is expected to result in a much lower (but *intra-vesicular*) encapsulation, as an example, Reshke et al. reported the loading of 1 siRNA per EV [84] in a very detailed article. However, this *intra-* versus *extra*-vesicular loading has demonstrated to be of a real importance. Proof of concept studies of siRNA delivery in vivo using EVs classified in these two categories lead to very different results: (i) siRNA loaded with electroporation (aggregated to EV) or EV coated with siRNA (lipid conjugated siRNA) need relatively high doses of siRNA to induce target knockdown (range 0.04 mg/kg [102] to 6 mg/kg [110], a dose range similar to the one needed for synthetic nanocarriers) whereas the only in vivo paper reporting EVs endogenously loaded with siRNA (i.e., *intra-vesicular*, using pre-miR 451 backbone) only need doses of about 10–300 ng/kg [84]. It seems therefore important to aim at loading EVs “intra-vesicularly” when loading EVs to benefit from their interesting delivery efficacy properties. Of note, using these endogenously/*intra*-vesicularly loaded EVs, an estimated human dose would then be ∼3 × 10^13^–9 ×10^14^ if calculated using a linear relation from mice to human and ∼2 × 10^12^–7 × 10^13^ if calculated taking using FDA guidelines [111] for dose selection based on murine data (12.3-fold reduction in dose/kg). The main barriers to large scale production of EVs depends on their origin: (i) EVs from ex vivo cell cultures are limited by the cost and technical difficulty associated with cell culture and the yield of EV production, (ii) EVs extracted from natural fluid (e.g., plasma) or cells (e.g., red blood cells) are less technically difficult and associated with a lower cost of production/sourcing compared to the cost of ex vivo cell culture at large scale. The main limits to large scale production in this case are the need to adapt to patient characteristics (e.g., ABO and HLA groups, etc.) and more importantly the cost associated with risks management for each patient derived small batch (viral and bacterial comtamination, blood group, etc.). Up to date, no clear data allow to decipher which EV parental cell would be more suited to perform drug delivery in general in terms of efficacy, although it is expected that EVs from immune-stimulatory cells (e.g., dendritic cells), xeno-origin or other “immune-detectable” EVs would probably be more prone to induce side effects and EVs from “stealth” cells like MSCs would be more suited.

The 10^12^–10^13^ dose range for a human dose is achievable in terms of production cost, allowing industrial transfer and subsequent medium to large scale patient treatment, this kind of technology may translate into EV therapeutics if our estimations are correct.

In terms of future improvement, achieving intra-vesicular loading (in order to keep the benefit of the natural EV endosomal escape properties) of ∼10% of the EV cytosol, a similar fraction of what is achieved with lipid nanoparticle (LNP) would already be very interesting for EV based therapeutics as it would already represent ∼6000 siRNA per EV. Of note, as it was reviewed and discuss recently [11,112], *intra-vesicular* loading (versus *aggregated* with EVs) is usually not reported, and various team advocated for a common data reporting frame for EV loading [11,112]. It is therefore difficult based on the literature to know the actual intra-vesicular loading that may be obtained by already described techniques. With a high loading, the theoretical delivery of only ∼2.5–25 EVs per liver cell, ∼0.75–7.5 EVs to each cell in the spleen or ∼18–180 EVs/cell in local administration (cf above) would probably be already sufficient to deliver a sufficient amount of an (si)RNA cargo of interest to achieve an effect. Lastly, systemic injections of highly loaded EVs may allow to reach intra-cytosolic doses at least similar to the one used in already approved systemic siRNA drugs like Patisiran (see Table 3), and combine the siRNA activity to the intrinsic biological activity of EVs, as for example, the anti-inflammatory properties of MSC-EVs. The estimation of engineered EVs’ cost tends to make it a relatively costly drug that would rather target life-threatening, resistant, orphan or intractable diseases. 

## 5. So What May Be “THE” EV Mechanism of Action?

The search for EV mechanism of action, as expected by regulatory agencies for drug approval, is still a partially answered question. The complexity of EVs assessed by omics tools like proteomics shows us that EV content is nearly as diverse as cellular ones. Therefore, the analogy with cell therapy’s mechanism of action may be of interest. As an example, mesenchymal stem cell (MSC) based cell therapies (from whom a large fraction of EVs potential drug products are derived) can hardly be explained by one specific pathway, and is now considered to be the result of a multifactorial effect involving both IDO-1 [115], PGE2, Nitric oxide [116], TGF-β1, IL-10, VEGF, HGF, mitochondrial transfer [117], etc. It is therefore very likely that MSC-EVs would also benefit from this pleiotropic effect as MSC-EVs are now reported to be the mediator of MSC therapeutic effect [118]. Some teams either tried to inhibit [3,6,7,119,120,121,122] or activate particular pathways [4] that were expected to be involved in the MSC-EV mechanism of action and have shown that in both cases the results were not unequivocal, each pathway having been only responsible for a fraction of the effect. 

Similarly, dendritic cell-derived EVs (Dexosomes) were expected to mediate an effect via MHC class II peptide presentation (a mechanism that prompted the use of the amount of MHC class II to standardize the injection dose in a clinical trial [123]), whereas MHC class II was later on shown not to be needed to achieve the dexosome effect [124], which is probably multifactorial. Apart from some limited and well-characterized EV therapeutic applications (e.g., EV-loaded enzyme replacement therapy [125], IL-12 intra-tumoral injection [126]), the EV mediated effect may rather difficultly be explained by a single pathway, although some other observation may help us to decipher which kind of pathway may be involved. 

As discussed above, an interesting observation is that EV exerts a large part of their effect in <60 min after their administration (and particularly in <20 min [36,37]). This kinetic is rather the one observed for receptor-ligand interactions, among which protein-based ones are the most common. The importance of protein was well demonstrated in immunomodulation models where treatment with protease nearly abrogated (∼70–80%) the EV effect [119], whereas treatment with triton X100 completely (∼100%) abrogated it, although membrane proteins lysis may also impact EV interaction with recipient cell endosomal membranes. Roefs et al. recently proposed a protein-based mechanism of action in tissue repair that may be of interest to the reader [127]. Of note, a particularly frequent mechanism of action reported involves the ERK1/2 and VEGF pathways. Although more prone to discussion, some more theoretical considerations may also prompt us to consider RNA-based information transfer via EVs as a mechanism with limited efficacy. Generally speaking, most information transfer between cells happens via ligand–receptor interaction. In the particular case of EVs, surface-to-volume considerations also tend to favor this mechanism. Indeed, if we consider cells to be about 10µm and EVs about 100 nm, the EV surface is “only” 10,000 smaller than a one of a cell whereas its volume is 1,000,000 times smaller. This 100 fold increase in surface-to-volume ratio in EVs compared to cells would make them good candidates for a ligand–receptor-based interaction with target cells rather than good intra-vesicular carriers. Knowing that proteins are the most known and described ligand–receptor mediators, a limited number of papers described the effect of other mediators like pro-resolving-lipids [128] or other potential lipophilic mediators that would be efficiently transported and presented to cells on EVs and are probably responsible for a fraction of MSC-EVs effect [129]. 

Finally, one may keep in mind that EV purification strategies may still largely be improved, as depicted by the very large range of the particle/microgram ratio (a widely reported purity marker) used in the published papers. EV preparations are therefore probably most of the time contaminated by protein aggregates and cytokines that may explain a large fraction of the EV effect, as was demonstrated by Whittaker et al. in a very interesting article [130]. Of note, contaminations may be reduced either by producing EVs in a serum or platelet lysate free media and/or by applying additional purification strategies, in particular via ultrafiltration or size exclusion chromatography.

## 6. Conclusions

Quantitative estimates from simplified pharmacokinetic and pharmacodynamic models based on quantitative data reported in the field suggest that information transfer through RNA by naturally circulating EVs may be limited in terms of efficacy at long distances. We suggest that if it exists, it may rather be at a short distance and/or in the reticulo-endothelial-system [24,84]. We consider like other authors [43] that ligand–receptor interactions are more prone to be used by EVs to transfer information (and interact with inflamed tissues or the brain–blood barrier) although the exact effectors (protein, lipids, others?) are probably very varied (and redundant?) depending on each biological process. On the other hand, these potential limitations of RNA transfer by native EV in physiological settings (and potentially in pathological conditions like cancer, inflammation) do not apply to EVs loaded with RNAs of interest for therapeutic purposes. Indeed, engineered EVs may benefit from the very interesting endosomal escape efficacy reported by some studies to deliver RNA molecules with much higher efficiency than synthetic vectors. Of note, to benefit from EVs’ outstanding delivery properties, RNA should probably be encapsulated within the vesicles without EV structure destruction and in sufficient amount to avoid the need for large EV doses that would be cost-prohibitive. Then, challenges faced for the clinical development of EV mediated RNA delivery concern both EV and RNA massive bio-production, as well as efficient encapsulation of RNA in EVs with controlled and scalable processes. Furthermore, EV based delivery may benefit from intrinsic EV properties depending on its parental cell type (angiogenesis [3], immunomodulation [4], increased cell proliferation [5], fibrosis inhibition [6], inflammation resolution [7], etc.) compared to synthetic vectors. Of note, quantitative results on which are based our estimations are usually very limited (if any) and subject to caution. Comparison with literature on other delivery systems is therefore difficult, and the field would highly benefit of a standardized data reporting frame. A particular caution should also be brought to common (and often unknown) purification or engineering artifacts before specifically attributing to EVs some observed effect. More generally, in our point of view more precautions should be taken for data interpretation in the EV domain, a young and interdisciplinary field with limited and difficult to interpret characterization methods. 

## Figures and Tables

**Table 1:** Summary of articles describing the transfer of RNAs via EVs as a major mechanism of EV mediated effect.

Author	Model/Context	Long/ Short Distance	Demonstration	Limits
Abels et al. [19]	Glioblastoma (GBM)	Short	Transfer of GBM EVs in 0.3% of microglial cells, presence of a GBM-miRNA in these cells	Partial (4/59) miRNA target induced silencing
			Injection of GBM EVs induce partial siRNA target knockdown	Highly supra-physiologic EV dose
Lucero et al. [20]	Glioblastoma (GBM)	Short	GBM EVs induce angiogenesis in vitro and a transcriptomic fingerprint is described	Supra physiologic dose (10^5^ EVs/cell)
			The transcriptomic fingerprint is found is also found in patients	There is only a correlation between EV treated GBM cells and patient GBM, no demonstration of causality is proposed
Shen et al. [21]	Tumor derived EVs	Short	Tumor derived EVs induce stemness in vitro	Supra physiologic dose (25:1 producing to receptor ratio)
			Limiting EV transfer in vivo diminish the effect on surrounding cells	KO of EV production in vivo is performed using a Rab-7 KO tumor model, this KO has a lot of other effects that may explain the difference observed
Ying et al. [22]	Glucose tolerance	Potentially both	miRNA is transferred from hematopoietic derived cells to liver cells in vivo	The transfer may be mediated by either EVs or Tunelling nanotubes (TNT) (and other?) mechanism, this miRNA being known to be transferred via TNT
Chen et al. [5]	Bone regeneration	Potentially both	MiR-375 is able to induce bone regeneration in vitro	No significant difference is observed compared to EVs not expressing miR-375 in vivo
Thomou et al. [24]	Transfer of miRNA from adipose tissue to liver	Potentially both	A serum-derived EV preparation transfers active miRNA to liver cells in vivo	The serum derived EV preparation purification protocol has a high chance to be comtaminated by extravesicular miRNA (up to 97.5% of miRNA purified)
Various teams [27,28,29]	CRE-mRNA transfer in vivo	Potentially both	CRE recombination is induced at long distance in the presence of EVs derived from cells expression CRE mRNA and protein	The CRE-Lox induced recombination may be mediated either by mRNA transfer via EVs but also or by transfer of mRNA or CRE protein by TNT, cell fusion, or extravesicular transfer
			A particular phenotype is described in CRE-recombined cells compared to non recombined cells	The causality in not demonstrated as a cell with a particular phenotype may be more prone to be transfected by CRE, in particular a more mobile and phagocytic cell. The sole endocytosis of nano-objects like EVs is also impacting the cell phenotype, even in the absence of cargo.

**Table 2:** Estimation of relevant parameters for a simplified extracellular vesicle (EV) pharmacokinetic modeling.

Parameter		Proposed Value	Reference
*Ctot* (*EV*)		10^12^ EV/L	[58,59]
*f* (*EV subtype*)	All EVs	100%	[60]
	Erythrocyte	4%	
	Platelet	51%	
	B cell	25.7%	
	CD4 cell	11%	
	All non hematopoietic tissue EVs	0.2%	
	Adipose tissue	0.16%	
	Other non hematopoietic tissue	0.04%	
Half life (τ½)		7 min (mice)	[61]
*f* (*target tissue*)	All tissues	100%	[57]
	Liver	60%	
	Spleen	15%	
	Lung	10%	
	Brain	0.5%	
*Nb Cell* (*tissue*)	All tissues	3.72 × 10^13^	[62]
	Liver	2.41 × 10^11^	
	Spleen	2 × 10^11^	
	Brain	3 × 10^12^	

**Table 3:** Comparison of key pharmacokinetic and delivery parameters of EVs and synthetic vectors (lipid nanoparticles, LNP). Of note, (?) depicts a very discussed or uncertain parameter, (*) number of nanoparticle per object is based on the following assumptions: 10:1 lipid to siRNA ratio [113,114], mean nanoparticle size 80 nm. ** As no EV-based therapeutics are on the market, cost estimations intervals are based on discussions with industrials in the EV field, comparison based on production costs and expected production-cost/final-cost ratio in the biotherapy field and typical reimbursement obtained for biotherapies in Europe and USA.

Variable	Naturally Circulating EVs	Therapeutic Use		
		Unmodified EVs	mi/siRNA Loaded/Engineered EVs	Synthetic siRNA Vectors Patisiran (LNP)
Total number of RNA per object	∼100	∼100	Up to 60,000 (?)	∼1000 (?) *
mi/siRNA of interest copy per object	Up to ∼0.1/EV	Up to ∼0.1/EV	∼1 [84]–60,000/EV (?)	∼1000/LNP (?) *
Delivery efficiency	∼20–30%	∼20–30%	∼3–30% [84] if intravesicular <1% if extravesicular [84] (?)	2% [114]
Half life	∼7 min	∼7 min	∼7 min (?)	3.2 days
Size	30–300 nm	30–300 nm	30–300 nm	60–100 nm
Typical dose injected	Daily production in blood estimated ∼4.3 × 10^14^ EVs Up to ∼4.3 × 10^13^ miRNA of interest	∼10^12^–10^13^ EVs Up to ∼10^12^ miRNA of interest	10^12^–10^13^ EVs (?) ∼10^12^–6 × 10^17^ (?) siRNA of interest	Systemic siRNA (patisiran) 0.3 mg/kg ∼1.3 × 10^16^ siRNA/kg ∼9.5 × 10^17^ siRNA/70 kg
Typical dose delivered in the cytosol	Up to ∼**9** × **10**^12^ miRNA of interest	Up to ∼**2** × **10**^11^ miRNA of interest	∼**2** × **10**^11^–**1.2** × **10**^16^ (?) siRNA of interest	∼**2** × **10**^15^ miRNA of interest
Typical expected cost for a dose	NA	∼**5000**–**25,000** **€** (?) **	∼**15,000**–**40,000** **€** (?) **	∼**13,000** **€**

## Data Availability

Not applicable.
